# Macular vessel density and foveal avascular zone parameters in patients after acute primary angle closure determined by OCT angiography

**DOI:** 10.1038/s41598-020-73223-9

**Published:** 2020-10-30

**Authors:** Kangcheng Liu, Huizhuo Xu, Haibo Jiang, Hua Wang, Pingbao Wang, Yi Xu, Fangling Li, Bei Xu, Xueyan Yao, Jing Zou

**Affiliations:** 1 Eye Center of Xiangya Hospital, Central South University, No. 87, Xiangya Road, Kaifu District, Changsha, 410008 Hunan China; 2 Hunan Key Laboratory of Ophthalmology, Changsha, 410008 Hunan China

**Keywords:** Diseases, Eye diseases, Signs and symptoms, Eye manifestations, Medical research, Biomarkers, Preclinical research, Biomarkers, Diagnostic markers, Predictive markers, Prognostic markers

## Abstract

This study analyzed the optical coherence tomography angiography (OCTA) macular parameters in primary angle-closure glaucoma (PACG) patients after acute primary angle closure (APAC) episodes. Thirty-three patients with 33 APAC eyes and 33 primary angle closure suspect (PACS) eyes and 33 age-matched normal subjects (controls) were enrolled. Macular vessel density (VD) in central, inner, outer and full regions and foveal avascular zone (FAZ) parameters (area, perimeter and circularity index) were compared between APAC, PACS, and control eyes. For resolved APAC eyes, the VD in each macular region was significantly lower than that in control eyes, with less central and inner macular VD than PACS eyes. The central macular VD was significantly lower in PACS eyes than in controls. There was no difference in FAZ area and perimeter between APAC, PACS, and control eyes. FAZ circularity was highest in control eyes, followed by PACS eyes, and lowest in APAC eyes. The AUC, sensitivity and specificity of FAZ circularity were 0.944, 93.9% and 84.8%, respectively, in APAC eyes and 0.881, 84.8% and 81.8%, respectively, in PACS eyes. Therefore, FAZ circularity had the best discrimination capability for detecting both APAC and PACS eyes. Macular assessment with OCTA could provide an accurate early-stage diagnostic tool for PACG.

## Introduction

Primary angle-closure glaucoma (PACG) is a notable type of glaucoma with high incidence in the population worldwide^1,2^. Though it is an important cause of blindness globally, the exact pathogenic mechanisms of PACG are not clear. Recently, some studies have indicated that the retinal vascular alterations are related to the development of this disease^3,4^.

At first reported in 2012, optical coherence tomography angiography (OCTA) is a safer technique to quantitatively assess retinal vasculature than conventional angiography methods^5–7^. A number of studies substantiated a significant decrease in peripapillary and macular vessel density (VD) in primary open angle glaucoma (POAG), and normal tension glaucoma (NTG) eyes compared to normal controls^8–10^. Some investigations^3,11^ focused on PACG patients and demonstrated that peripapillary VD was lower in acute PACG eyes than in unaffected eyes after an acute primary angle closure attack^12,13^. In addition, PACG eyes with well-controlled intraocular pressure (IOP) had higher peripapillary VD than eyes with IOP that was not well controlled^14^.

Some studies reported macular damage in early-stage glaucoma, such as macular ganglion cell-inner plexiform layer loss, and these attracted a significant amount of research to understand the importance of the macula in glaucoma^15,16^. Regarding anatomy, the peripapillary area is supported by a double-layered capillary support system (the retinal nerve fiber layer (RNFL) and ganglion cell layer (GCL)), while the vascular supply of the macula is only a single-layered parafoveal capillary arcade. Physiological evidence also supports that the macula is more sensitive to hypoxia and ischaemia because it consumes more oxygen than other tissues in the body^17^. Subsequently, macular VD and foveal avascular zone (FAZ) metrics were regarded as potential biomarkers in the clinical evaluation of early glaucoma in recent studies^18–20^. However, few researches have examined macular vasculature in PACG. Zhu et al.^14^ demonstrated decreased macular circulation in PACG with glaucomatous optic neuropathy and visual field (VF) defects, but no literature has explored macular VD and FAZ metrics using OCTA in APAC eyes after a recent acute episode. The purpose of our study was to investigate the potential macular vascular differences with OCTA by comparing macular VD and FAZ parameters between APAC eyes after the first acute attack, fellow eyes confirmed to be affected with primary angle closure suspect (PACS) and heathy normal control eyes.

## Methods

### Subjects

A total of 33 unilateral adult APAC patients (33 APAC eyes and 33 PACS eyes) who were diagnosed as acute angle-closure glaucoma patients were enrolled in the study, and 33 age-matched healthy subjects were enrolled as controls, with 33 eyes randomly selected, from June 2018 to December 2019 at the Department of Ophthalmology of Xiangya Hospital. All research methods follow the Helsinki Declaration. After approval by the Medical Ethics Committee of Xiangya Hospital, all the subjects signed an informed consent form according to the principle of ethics.

Eyes that suffered the first attack and were resolved by treatment with medicine were designated as APAC eyes, and the contralateral unaffected eyes were designated as PACS eyes. During hospitalization the APAC eyes underwent trabeculectomy, and the PACS eyes were treated with laser peripheral iridotomy. An APAC eye was defined by the presence of the following^21^: (1) elevated IOP > 21 mmHg with Goldmann applanation tonometry; (2) at least 3 of the following signs: conjunctival hyperaemia, corneal epithelial edema, enlarged pupil, direct disappearance of light reflex, and the disappearance of the shallow anterior chamber or anterior chamber; (3) at least two of the following symptoms: severe eye pain, a sharp decline in vision, ipsilateral migraine, nausea, vomiting, elevated body temperature, and pulse acceleration.; and(4) at least 2 quadrants of the chamber angle were closed on gonioscopic examination. A PACS eye was defined by the presence of the following^22^: (1) presence of iridotrabecular contact more than 180° without peripheral anterior synechiae (PAS) on gonioscopy; (2) the absence of a glaucomatous optic nerve and visual field damage; (3) no history or signs of a previous AAC attack; and (4) an IOP of < 21 mmHg without medication. Exclusion criteria for APCG and PACS patients were as follows: (1) secondary glaucoma, such as lens-derived glaucoma, neovascular glaucoma, iridocyclitis secondary glaucoma; (2) POAG, chronic angle-closure glaucoma, congenital or developmental glaucoma; (3) retinal or optic nerve disease; (4) dioptre spherical degree > 6.00–6.00 D and/or astigmatism > 3.00–3.00 D; (5) previous history of endoscopic or laser treatment; and (6) history of hypertension and diabetes.

The control subjects were excluded if they met the following criteria: (1) ophthalmic diseases such as glaucoma, fundus diseases, corneal diseases; (2) history of ocular trauma and intraocular surgery, history of hypertension and diabetes, family history of glaucoma; (3) retinal or optic nerve diseases or visual field defect; (4) dioptre spherical power > 6.00 –6.00 D and / or astigmatism > 3.00–3.00 D (5) IOP was higher than 21 mmHg; and (6) a cup-to-disc ratio ≥ 0.5 or cup to disc asymmetry ≥ 0.2.

### Ophthalmic examination

We used anti-glaucoma medicine and intravenous acetazolamide or mannitol to reduce IOP for all APAC eyes after admission. A series of ophthalmologic examinations were performed in APAC and PACS eyes after the first acute attack had been fully resolved by medicine. Subsequently, the APAC eyes underwent trabeculectomy, and the PACS eyes were treated with laser peripheral iridotomy during hospitalization.

The complete ophthalmologic examinations included vision examination, slit lamp examination, gonioscopic examination, IOP measurement (Goldmann applanation tonometry), anterior chamber depth (ACD) and axial length (AL) measurements, field visual measurements, and OCT and OCTA examinations. We used OCT (Carl Zeiss Meditec, Dublin, California, USA) to measure RNFL thickness and used a Humphrey Field Analyser (Zeiss Humphrey Systems, Dublin, California, USA) for field visual inspection. Measurements of IOP, systolic blood pressure (SBP), and diastolic blood pressure (DBP) were performed while the patients was in a sitting position for 20 min before the patient underwent the OCTA examination. The mean arterial pressure (MAP) was calculated by the following formula: MAP = DBP + (SBP − DBP)/3, the ocular perfusion pressure (OPP) calculation formula was OPP = 2 (MAP − IOP)/3^23^.

### Optical coherence tomography angiography

**Figure 1:**
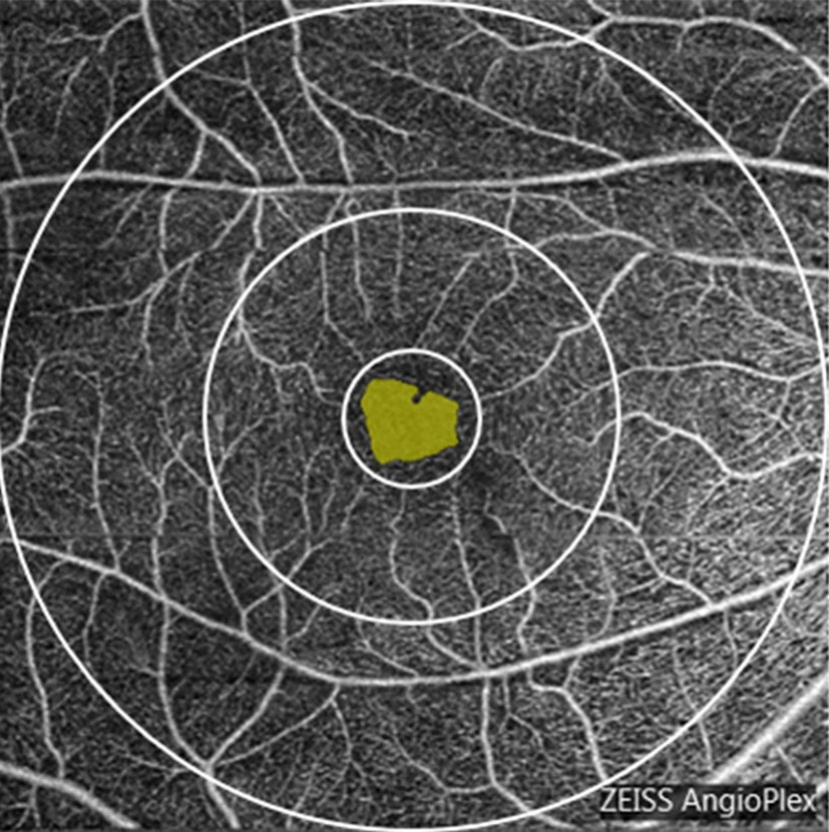
Macular area measurement map, the central area is the center 1 mm diameter range, the inner ring and the outer ring are respectively 3 mm, 6 mm diameter range. The yellow zone is foveal avascular zone.

All subjects underwent SD-OCTA examination using the OCTA system (Cirrus; Zeiss, Dublin, USA; software version 10.0.0.14618). OCTA obtained a 6 × 6 mm volumetric macular superficial retinal vessel image that consisted of vessels from the layer of the inner limiting membrane (ILM) to the inner plexiform layer (IPL). Several factors, including glaukomaflecken and cataract, can affect VD measurements in the macula. Therefore, poor-quality scans with a signal strength index (SSI) values less than 7 were excluded from this study. The system software was used to automatically divide the image into 3 regions, including a 1 mm diameter central disc and an inner and outer ring with diameters of 3 mm and 6 mm, respectively (Fig. 1). The blood vessel length density (VLD, defined as the total length of perfused vasculature) and vessel perfusion density (VPD, defined as the total area of perfused vasculature per unit area in a region of measurement), which were assessed with high repeatability and reproducibility, were calculated separately at various distances from the fovea: central (1 mm diameter region), inner (1–3 mm diameter region), outer (3–6 mm diameter region) and full (6 mm diameter region)^24^.

The FAZ indicators of the macular area, including area, perimeter and circularity index, were automatically calculated by the system software (Cirrus; Zeiss, Dublin, USA; software version 10.0.0.14618) (Fig. 1). The FAZ circularity index is a measure of the shape of the FAZ, where 1 indicates a circular shape and 0 indicates an irregular shape. By observing the parameters of the FAZ, we can understand the damage that the disease to inflicts upon the capillaries in the macular arch area. The FAZ circularity index calculation method was based on the area and perimeter of the FAZ, and the calculation formula is as follows^19^:

$$\frac{{Area}_{FAZ}}{\pi {\left(\frac{{Perimeter}_{FAZ}}{2\pi }\right)}^{2}}=\frac{{Area}_{FAZ}}{\left(\frac{{{Perimeter}^{2}}_{FAZ}}{4\pi }\right)}=\frac{{4\pi Area}_{FAZ}}{{{Perimeter}^{2}}_{FAZ}}$$

### Statistical analysis

Statistical analysis was performed with SPSS version 20.0 (SPSS Inc, Chicago, IL, USA). The normal distribution of the data was tested by the Kolmogorov–Smirnov test. Therefore, the data are presented as the mean ± standard deviation (range) of the Gaussian distribution value and the median (95% confidence interval) of the non-Gaussian distribution. The comparison between two different groups was analysed with the LSD-t test with Bonferroni correction for Gaussian distribution values and Tamhane’s T2 test with Bonferroni correction for non-Gaussian distribution values, and *P* < 0.0167 was considered statistically significant. The discrimination capability of OCTA parameters were evaluated with the area under the receiver operating characteristic curve (AUROC), and *P* < 0.05 was considered statistically significant. Multiple linear regression analyses were conducted to evaluate the relationship between OCTA parameters and other clinical features in the APAC and PACS eyes. To further evaluate these co-factors for macular VD, we established regression models for predicting macular VD based on the clinical features.

## Results

### Clinical features and parameters of the participants

There were 33 PACG patients, including those who had both eyes as APAC eyes and PACS eyes, and 33 age-matched normal subjects in this study.

Table 1 summarizes the clinical data of all subjects and their comparison results. There was no significant difference in age, SSI, IOP or visual acuity at imaging, RNFL thickness or OPP between APAC, PACS and control eyes (*P* > 0.0167). Compared with control eyes, APAC eyes and PACS eyes all had shorter AL (*P* < 0.001); there was no difference in AL between APAC and PACS eyes. The ACD of APAC eyes (1.74 (1.49–1.86) mm) was significantly lower than that of PACS (1.90 (1.80–2.00) mm) (*P* < 0.001) and control eyes (2.86 (2.63–2.99) mm) (*P* < 0.001); the ACD of PACS eyes was significantly lower than that of control eyes (*P* < 0.001). The VFof APAC group (− 8.92 (− 11.81 to − 7.14) dB) was significantly lower than that of PACS group (− 3.85 (− 5.34 to − 2.31) dB) (*P* < 0.001) and control group (− 3.47 (− 3.92 to − 2.89) dB) (*P* < 0.001). For APAC eyes, the average IOP at attacking was 52.00 (42.00–58.00) mmHg, and the duration of IOP exposure was 2.35 ± 1.50 days. There was no significant difference in IOP at attack and VF between PACS eyes and control eyes (*P* > 0.0167). The SBP values of APAC and PACS eyes were significantly higher than that of control eyes (*P* = 0.001), but the DBP values of APAC and PACS eyes were significantly lower than that of control eyes (*P* = 0.003).

**Table 1:** Clinical features and parameters of the participants.

Variables	APAC (n = 33)	PACS (n = 33)	Control (n = 33)	APAC vs PACS	APAC vs Ctr	PACS vs Ctr
Sex (M/F)	13/20	13/20	13/20	/	/	/
SSI	9 (8, 10)	10 (8, 10)	10 (9, 10)	/	/	/
Age (years)*	60.58 ± 8.36	60.58 ± 8.36	59.61 ± 5.41	/	0.601*	0.601*
ACD (mm)^T^	1.74 (1.49, 1.86)	1.90 (1.80, 2.00)	2.86 (2.63, 2.99)	*P* < 0.001^T^	*P* < 0.001^T^	*P* < 0.001^T^
AL (mm)^T^	21.78 (21.39, 22.23)	21.87 (21.49, 22.38)	23.31 (22.54, 23.71)	0.937^T^	*P* < 0.001^T^	*P* < 0.001^T^
Duration time (days)	2.35 ± 1.50	/	/	/	/	/
IOP at attack(mmHg) ^T^	52.00 (42.00–58.00)	15.00 (14.00–17.00)	15.00 (13.00–17.00)	*P* < 0.001^T^	*P* < 0.001^T^	0.993^T^
IOP at imaging(mmHg) ^T^	14.00 (11.00, 17.00)	15.00 (13.00–17.00)	15.00 (13.00–17.00)	0.277^T^	0.533^T^	0.936^T^
Visual acuity at imaging (LogMAR) ^T^	0.30 (0.22, 052)	0.10 (0.00, 0.30)	0.15 (0.05, 0.22)	*P* < 0.001^T^	*P* < 0.001^T^	0.974^T^
RNFL thickness (μm) ^T^	98.00 (81.00–106.00)	99.00 (95.00–105.00)	99.00 (96.00–105.00)	0.227^T^	0.207^T^	1.000^T^
VFMD (dB) ^T^	− 8.92 (− -11.81 to − 7.14)	− 3.85 (− 5.34 to − 2.31)	− 3.47 (− 3.92 to − 2.89)	*P* < 0.001^T^	*P* < 0.001^T^	0.233^T^
SBP (mmHg) ^T^	133 (130, 138)	133 (130, 138)	126 (117, 129)	/	0.001^T^	0.001^T^
DBP (mmHg)*	76.03 ± 7.17	76.03 ± 7.17	80.79 ± 4.68	/	0.003*	0.003*
OPP (mmHg)*	53.41 ± 4.62	52.50 ± 3.89	53.39 ± 3.82	0.371*	0.879*	0.458*

*Ctr* control, *APAC* acute primary angle closure, *PACS* primary angle closure suspect, *SSI* signal strength index, *ACD* anterior chamber depth, *AL* axial length, *IOP* intraocular pressure, *RNFL* retina nerve fiber layer, *SBP* systolic blood pressure, *DBP* diastolic blood pressure, *VFMD* visual field mean deviation, *OPP* ocular perfusion pressure.

^T^(Tamhane’s T2 test).

*(LSD-t test).

### OCTA parameters of the participants

Compared with PACS eyes, VLD and VPD values of APAC eyes were significantly decreased in central and inner macular regions, with statistically significant differences (*P* ≤ 0.015), though there was still no significant change in the outer and full macular regions (*P* ≥ 0.025). In APAC eyes, VLD and VPD values were significantly reduced in all macular regions compared with control eyes, with statistically significant differences (*P* ≤ 0.001). VLD and VPD values in the central macular region of PACS eyes were significantly lower than those of control eyes (*P* ≤ 0.004), while the differences in other regions were not statistically significant (*P* ≥ 0.032). For the FAZ index, there was no difference in the FAZ area and perimeter between the three groups (*P* ≥ 0.018). The FAZ circularity index was highest in control eyes (0.76 ± 0.07), followed by PACS eyes (0.61 ± 0.11), and lowest in APAC eyes (0.52 ± 0.12) (*P* ≤ 0.007) (Table 2).

**Table 2:** OCTA parameters of the participants.

VLD	APAC (n = 33)	PACS (n =33)	Control (n = 33)	APAC vs PACS	APAC vs Ctr	PACS vs Ctr
Central	4.92 ± 2.36	6.25 ± 2.28	7.89 ± 1.89	0.015	*P* < 0.001^T^	0.003
Inner	14.09 ± 3.56	16.6 ± 2.14	17.66 ± 1.72	0.003	*P* < 0.001^T^	0.086
Outer	14.93 ± 3.46	16.61 ± 2.21	17.73 ± 1.36	0.066	*P* < 0.001^T^	0.048
Full	14.47 ± 3.30	16.32 ± 2.06	17.44 ± 1.39	0.025	*P* < 0.001^T^	0.037

VPD	APAC	PACS	Control	APAC vs PACS	APAC vs Ctr	PACS vs Ctr
Central	0.09 ± 0.05	0.13 ± 0.06	0.18 ± 0.04	0.015	*P* < 0.001^T^	0.004
Inner	0.34 ± 0.09	0.39 ± 0.05	0.42 ± 0.05	0.012	*P* < 0.001^T^	0.052
Outer	0.37 ± 0.08	0.41 ± 0.06	0.44 ± 0.04	0.064	*P* < 0.001^T^	0.051
Full	0.35 ± 0.09	0.4 ± 0.05	0.43 ± 0.04	0.026	*P* < 0.001^T^	0.032

FAZ	APAC	PACS	Control	APAC vs PACS	APAC vs Ctr	PACS vs Ctr
Area	0.27 ± 0.14	0.29 ± 0.15	0.26 ± 0.09	0.919	0.978	0.669
Perimeter	2.53 ± 0.86	2.39 ± 0.62	2.06 ± 0.40	0.843	0.018	0.033
Circularity	0.52 ± 0.12	0.61 ± 0.11	0.76 ± 0.07	0.007	*P* < 0.001^T^	*P* < 0.001^T^

*Ctr* control, *APAC* acute primary angle closure, *PACS* primary angle closure suspect, *SSI* signal strength index, *VLD* vessel length density, *VPD* vessel perfusion density, *FAZ* foveal avascular zone.

### Receiver-operating characteristic curve

**Figure 2:**
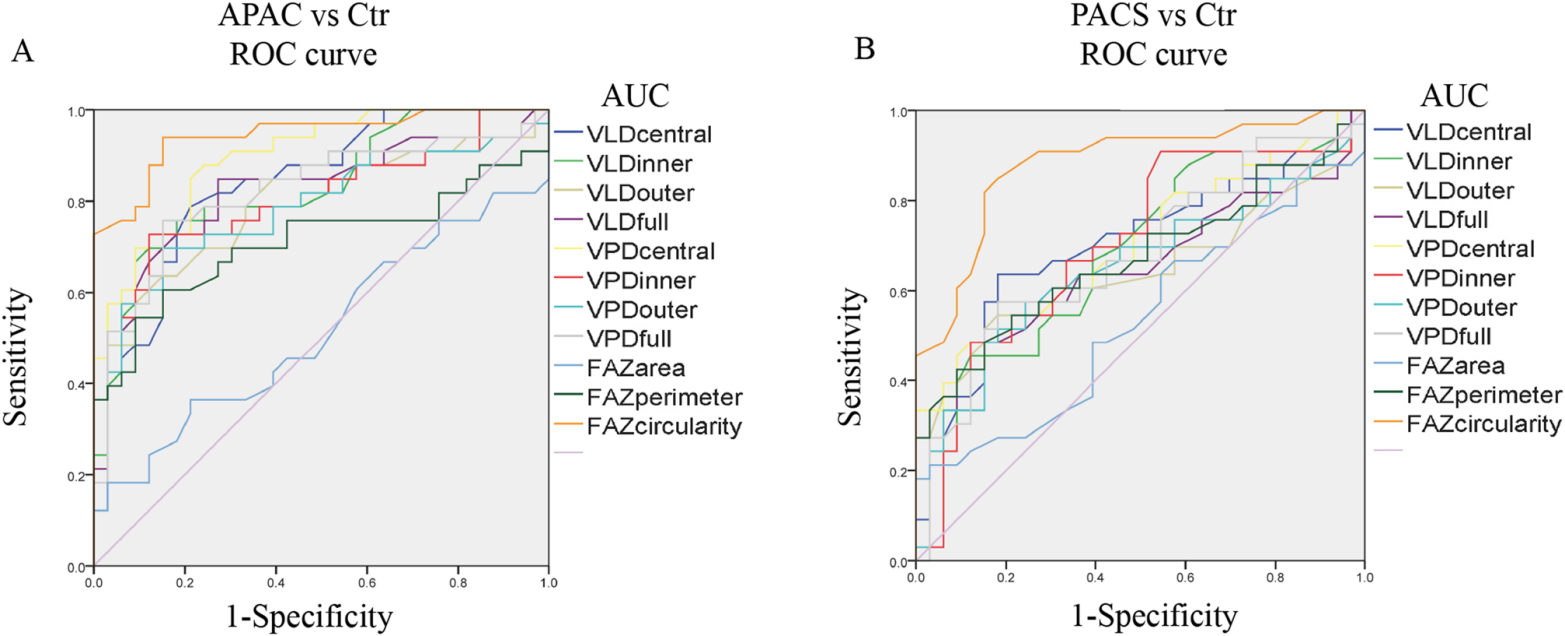
ROC curve analysis of macular vessel density and foveal avascular zone parameters between (**A**) APAC and control eyes, and (**B**) PACS and control eyes. *Ctr* control, *APAC* acute primary angle closure, *PACS* primary angle closure suspect, *SSI* signal strength index, *VLD* vessel length density, *VPD* vessel perfusion density, *FAZ* foveal avascular zone, *AUC* area under the receiver operating characteristic curve.

We obtained different OCTA partition parameters between the APAC group, PACS group and control group, which might be used as markers for identifying APAC, PACS and healthy control eyes. To test this possibility, different OCTA parameter values were used to analyze the ROC curve and calculate the area under the curve (AUC) to evaluate the accuracy of detection (Fig. 2). The AUC for discriminating APAC eyes from normal eyes was highest with the FAZ circularity index (0.944), followed by the central VPD (0.897) and central VLD (0.840). Compared with other macular VD parameters, the FAZ circularity index exhibited better sensitivity (0.939) and specificity (0.848) in APAC eyes. The rank of the indexes for distinguishing PACS eyes from normal eyes was the FAZ circularity index (0.881), central VLD (0.704) and central VPD (0.704). In addition, both the sensitivity and specificity of the FAZ circularity index reached 80% simultaneously in PACS eyes (Table 3).

**Table 3:** The areas under the ROC curve (AUC) for results.

	APAC vs Ctr				PACS vs Ctr			
	AUC	*P*	Sensitivity	Specificity	AUC	*P*	Sensitivity	Specificity
**VLD**								
Central	0.840	*P* < 0.001	0.788	0.788	0.704	0.004	0.636	0.818
Inner	0.831	*P* < 0.001	0.758	0.818	0.678	0.013	0.667	0.606
Outer	0.791	*P* < 0.001	0.636	0.848	0.638	0.054	0.545	0.818
Full	0.819	*P* < 0.001	0.848	0.727	0.648	0.039	0.485	0.879
**VPD**								
Central	0.897	*P* < 0.001	0.879	0.758	0.704	0.004	0.455	0.909
Inner	0.798	*P* < 0.001	0.727	0.879	0.697	0.006	0.485	0.879
Outer	0.784	*P* < 0.001	0.697	0.848	0.652	0.034	0.515	0.818
Full	0.820	*P* < 0.001	0.758	0.848	0.680	0.012	0.576	0.818
**FAZ**								
Area	0.515	0.832	0.364	0.788	0.534	0.635	0.212	0.970
Perimeter	0.711	0.003	0.606	0.848	0.672	0.016	0.424	0.909
Circularity	0.944	*P* < 0.001	0.939	0.848	0.881	*P* < 0.001	0.848	0.818

*Ctr* control, *APAC* acute primary angle closure, *PACS* primary angle closure suspect, *ROC* receiver-operating characteristic, *VLD* vessel length density, *VPD* vessel perfusion density, *FAZ* foveal avascular zone.

### Correlation analysis of OCTA parameters and other clinical features and parameters of the participants

For the APAC group, the outer and full VLD were positively correlated with the RNFL, with statistically significant differences (r = 0.373, *P* = 0.032; r = 0.473, *P* = 0.005); the central VLD was positively correlated with the VF and SBP (r = 0.449, *P* = 0.009; r = 0.426, *P* = 0.013). The outer and full VPD were positively correlated with the RNFL thickness significantly (r = 0.462, *P* = 0.007; r = 0.461, *P* = 0.007); the FAZ area and circularity index were positively correlated with the RNFL thickness significantly (r = 0.474, *P* = 0.005; r = 0.361, *P* = 0.039) (Table 4).

**Table 4:** Summary of univariate regression analyses OCTA parameters and other clinical features and parameters of the APAC participants.

Clinical parameter	Central		Inner		Outer		Full	
	*r (ρ)*	*p*	*r (ρ)*	*p*	*r (ρ)*	*p*	*r (ρ)*	*p*
**VLD**								
Age	0.203	0.257	− 0.094	0.604	− 0.232^$^	0.194^$^	− 0.121	0.504
ACD	− 0.152	0.399	0.047	0.796	0.035^$^	0.848^$^	0.016	0.931
AL	− 0.071	0.696	0.006	0.973	− 0.010^$^	0.955^$^	− 0.008	0.966
IOP at attack	0.011	0.953	− 0.091	0.614	− 0.124^$^	0.491^$^	− 0.104	0.564
IOP at imaging	− 0.004	0.983	0.111	0.540	0.165^$^	0.360^$^	0.139	0.440
RNFL thickness	0.184	0.305	0.294	0.097	0.373*^$^	0.032^$^	0.473*	0.005
VFMD	0.449*	0.009	0.239	0.181	0.243^$^	0.172^$^	0.186	0.300
SBP	0.426*^$^	0.013^$^	0.147^$^	0.416^$^	− 0.060^$^	0.741^$^	− 0.005^$^	0.978^$^
DBP	− 0.117	0.517	− 0.036	0.842	− 0.152^$^	0.398^$^	− 0.094	0.601
OPP	0.045	0.804	− 0.069	0.704	− 0.235^$^	0.188^$^	− 0.178	0.322
**VPD**								
Age	0.005	0.978	− 0.057	0.752	− 0.149	0.409	− 0.054	0.764
ACD	− 0.101	0.574	0.109	0.544	0.004	0.985	− 0.024	0.895
AL	0.042	0.816	0.020	0.911	− 0.004	0.983	0.002	0.993
IOP at attack	− 0.035	0.848	− 0.139	0.440	− 0.054	0.765	− 0.091	0.613
IOP at imaging	− 0.148	0.411	0.075	0.679	0.241	0.177	0.086	0.634
RNFL thickness	0.021	0.909	0.103	0.567	0.462*	0.007	0.461*	0.007
VFMD	0.246	0.167	0.197	0.271	0.218	0.222	0.218	0.224
SBP	0.106^$^	0.558^$^	0.219^$^	0.221^$^	− 0.059^$^	0.746^$^	− 0.016^$^	0.928^$^
DBP	− 0.248	0.163	− 0.013	0.945	− 0.172	0.340	− 0.189	0.292
OPP	− 0.097	0.591	− 0.019	0.916	− 0.310	0.080	− 0.219	0.220

FAZ	Area		Perimeter		Circularity			
	*r (ρ)*	*p*	*r (ρ)*	*p*	*r (ρ)*	*p*		
Age	− 0.121	0.504	0.112	0.536	0.239	0.180		
ACD	0.016	0.931	− 0.175	0.330	− 0.195	0.277		
AL	− 0.008	0.966	0.088	0.627	0.049	0.787		
IOP at attack	− 0.104	0.564	0.104	0.566	0.155	0.388		
IOP at imaging	0.139	0.440	0.155	0.388	0.179	0.319		
RNFL thickness	0.473*	0.005	0.309	0.080	0.361*	0.039		
VFMD	0.186	0.300	0.122	0.499	0.051	0.778		
SBP	− 0.069^$^	0.703^$^	0.034^$^	0.851^$^	− 0.176^$^	0.327^$^		
DBP	− 0.094	0.601	− 0.093	0.606	− 0.084	0.643		
OPP	− 0.178	0.322	− 0.215	0.230	− 0.168	0.349		

Gaussian distribution data was analyzed by Pearson correlation analysis. Non-Gaussian distribution data was analyzed by Spearman's rank correlation analysis. r is a measure of the intensity of the effect in Pearson 's correlation coefficient and ρ is a measure of the intensity of the effect in Spearman's correlation coefficient.

^$^(Spearman’s correlation).

*(*p* < 0.05).

*APAC* acute primary angle closure, *ACD* anterior chamber depth, *VLD* vessel length density, *VPD* vessel perfusion density, *FAZ* foveal avascular zone, *AL* axial length, *IOP* intraocular pressure, *RNFL* retina nerve fiber layer, *SBP* systolic blood pressure, *DBP* diastolic blood pressure, *VFMD* visual field mean deviation.

For PACS group, the outer and full VPD were positively correlated with the RNFL thickness (r = 0.378, *P* = 0.030; r = 0.356, *P* = 0.042), DBP (r = 0.347, *P* = 0.048; r = 0.348, *P* = 0.048), and OPP (r = 0.359, *P* = 0.040; r = 0.380, *P* = 0.029) significantly; the inner VPD was positively correlated with OPP (r = 0.384, *P* = 0.027). The FAZ area and perimeter index were positively correlated with DBP (r = 0.417, *P* = 0.016; r = 0.423, *P* = 0.014) and OPP (r = 0.551, *P* = 0.001; r = 0.649, *P* = 0.001) significantly (Table 5).

**Table 5:** Summary of univariate regression analyses OCTA parameters and other Clinical features and parameters of the PACS participants.

Clinical parameter	Central		Inner		Outer		Full	
	r (ρ)	*p*	r (ρ)	*p*	r (ρ)	*p*	r (ρ)	*p*
**VLD**								
Age	0.140	0.438	0.329	0.062	0.264	0.137	0.293	0.098
ACD	− 0.267	0.133	− 0.280	0.115	− 0.193	0.283	− 0.229	0.201
AL	0.016^$^	0.928^$^	− 0.096^$^	0.594^$^	0.061^$^	0.735^$^	0.017^$^	0.926^$^
IOP at attack	0.055	0.763	− 0.060	0.740	− 0.051	0.776	− 0.054	0.764
IOP at imaging	− 0.103	0.567	0.008	0.966	− 0.066	0.716	− 0.053	0.770
RNFL thickness	− 0.127	0.481	− 0.323	0.067	− 0.171	0.342	− 0.217	0.225
VFMD	− 0.044	0.808	0.023	0.901	0.162	0.368	0.137	0.447
SBP	− 0.169^$^	0.348^$^	− 0.051^$^	0.778^$^	− 0.083^$^	0.647^$^	− 0.078^$^	0.666^$^
DBP	− 0.255	0.153	− 0.022	0.905	− 0.119	0.510	− 0.104	0.564
OPP	− 0.226	0.205	0.003	0.987	− 0.082	0.649	− 0.069	0.704
**VPD**								
Age	− 0.027	0.883	0.054	0.764	0.016	0.929	0.024	0.894
ACD	0.078	0.666	− 0.006	0.973	0.054	0.767	0.046	0.799
AL	0.095^$^	0.598^$^	− 0.050^$^	0.781^$^	0.122^$^	0.498^$^	0.113^$^	0.531^$^
IOP at attack	− 0.103	0.569	− 0.022	0.905	− 0.049	0.785	− 0.050	0.781
IOP at imaging	0.039	0.831	− 0.047	0.795	0.124	0.492	0.094	0.604
RNFL thickness	0.133	0.462	0.184	0.305	0.378*	0.030	0.356*	0.042
VFMD	0.113	0.530	− 0.058	0.750	0.144	0.425	0.107	0.552
SBP	0.005^$^	0.977^$^	0.281^$^	0.113^$^	0.222^$^	0.215^$^	0.224^$^	0.210^$^
DBP	0.058	0.749	0.273	0.125	0.347*	0.048	0.348*	0.048
OPP	0.034	0.850	0.384*	0.027	0.359*	0.040	0.380*	0.029

FAZ	Area		Perimeter		Circularity			
	r (ρ)	*p*	r (ρ)	*p*	r (ρ)	*p*		
Age	− 0.210^$^	0.242^$^	− 0.042	0.815	− 0.289	0.103		
ACD	− 0.327^$^	0.063^$^	− 0.296	0.095	− 0.013	0.943		
AL	0.030^$^	0.867^$^	0.000^$^	0.999^$^	− 0.027^$^	0.882^$^		
IOP at attack	0.293^$^	0.098^$^	0.276	0.120	− 0.037	0.839		
IOP at imaging	− 0.175^$^	0.330^$^	− 0.313	0.076	0.150	0.404		
RNFL thickness	0.317^$^	0.072^$^	0.240	0.178	0.067	0.712		
VFMD	0.075^$^	0.679^$^	0.210	0.240	− 0.295	0.095		
SBP	0.272^$^	0.126^$^	0.335^$^	0.056^$^	0.050^$^	0.780^$^		
DBP	0.417*^$^	0.016^$^	0.423*	0.014	0.079	0.662		
OPP	0.551*^$^	0.001^$^	0.649*	< 0.001	− 0.053	0.771		

Gaussian distribution data was analyzed by Pearson correlation analysis. Non-Gaussian distribution data was analyzed by Spearman's rank correlation analysis. r is a measure of the intensity of the effect in Pearson’s correlation coefficient and ρ is a measure of the intensity of the effect in Spearman's correlation coefficient.

*PACS* primary angle closure suspect, *ACD* anterior chamber depth, *VLD* vessel length density, *VPD* vessel perfusion density, *FAZ* foveal avascular zone, *AL* axial length, *IOP* intraocular pressure, *RNFL* retina nerve fiber layer, *SBP* systolic blood pressure, *DBP* diastolic blood pressure, *VFMD* visual field mean deviation.

^$^(Spearman’s correlation).

*(*p* < 0.05).

Additionally, we used multivariate stepwise regression analyses to further evaluate the co-factors for macular VD (Table 6). For the APAC group, we could predict changes in central VLD by looking at changes in VF (*P* = 0.005) and SBP (*P* = 0.037). The determination coefficient R^2^ = 0.265 indicated that 26.5% of the central VLD variation may have been attributed to the joint variations in VF and SBP.

**Table 6:** Predictors of macular VD by multiple linear regression analysis in APAC and PACS eyes.

Group	Parameter	Regression formula	Adjusted *R*^2^	Durbin-Watson	*p*
APAC	VLD central	$- \;{5}.{626 } + \, 0.{\text{239VFMD }} + \, 0.0{\text{97SBP}}$(1)	0.265	1.831	0.004
PACS	VPD outer	$- \;0.{142} + 0.00{\text{3RNFL}} + 0.00{\text{1DBP}} + 0.00{\text{4OPP}}$ (2)	0.182	1.167	0.032
	VPD full	$- \;0.{1}0{8} + 0.00{\text{2RNFL}} + 0.00{\text{DBP}} + 0.00{\text{4OPP}}$ (3)	0.179	1.197	0.033
	FAZ area	$- \;0.{883} - 0.00{\text{1DBP}} + 0.0{\text{24OPP}}$(4)	0.317	2.169	0.001
	FAZ perimeter	$- \;{3}.0{74} - 0.0{\text{23DBP}} + 0.{\text{137OPP}}$(5)	0.409	1.866	< 0.001

*APAC* acute primary angle closure, *PACS* primary angle closure suspect, *VLD* vessel length density, *VPD* vessel perfusion density, *FAZ* foveal avascular zone, *AL* axial length, *IOP* intraocular pressure, *RNFL* retina nerve fiber layer, *SBP* systolic blood pressure, *DBP* diastolic blood pressure, *OPP* ocular perfusion pressure, *VFMD* visual field mean deviation.

For the PACS group, Eq. (2) shows that we could predict the outer VPD by looking at changes in the RNFL thickness (*P* = 0.039), DBP (*P* = 0.713), and OPP (*P* = 0.342). The determination coefficient R^2^ = 0.182 indicated that 18.2% of the outer VPD variation may have been attributed to joint variations in RNFL thickness, DBP and OPP. Equation (3) shows that we could predict changes in the full VPD by looking at changes in RNFL thickness (*P* = 0.051), DBP (*P* = 0.831), and OPP (*P* = 0.250). The determination coefficient R^2^ = 0.179 indicated that 17.9% of the full VPD variation may have been attributed to joint variations in RNFL thickness, DBP and OPP. Although RNFL thickness, DBP and OPP had combined effects on the outer and full VPD, RNFL thickness had a larger impact on VPD variations than DBP and OPP in PACS eyes.

Equation (4) shows that we could predict changes in the FAZ area by looking at changes in DBP (*P* = 0.871) and OPP (*P* = 0.014). The determination coefficient R^2^ = 0.317 indicated that 31.7% of the FAZ area variation may have been attributed to joint variations in DBP and OPP. Equation (5) shows that we could predict changes in the FAZ perimeter by looking at changes in DBP (*P* = 0.256) and OPP (*P* = 0.001). The determination coefficient R^2^ = 0.409 indicated that 40.9% of the FAZ perimeter variation may have been attributed to joint variations in DBP and OPP. These two multi-variate regression models presented that OPP was a more important factor than DBP for FAZ area and perimeter measurements in PACS eyes.

### Representative cases

**Figure 3:**
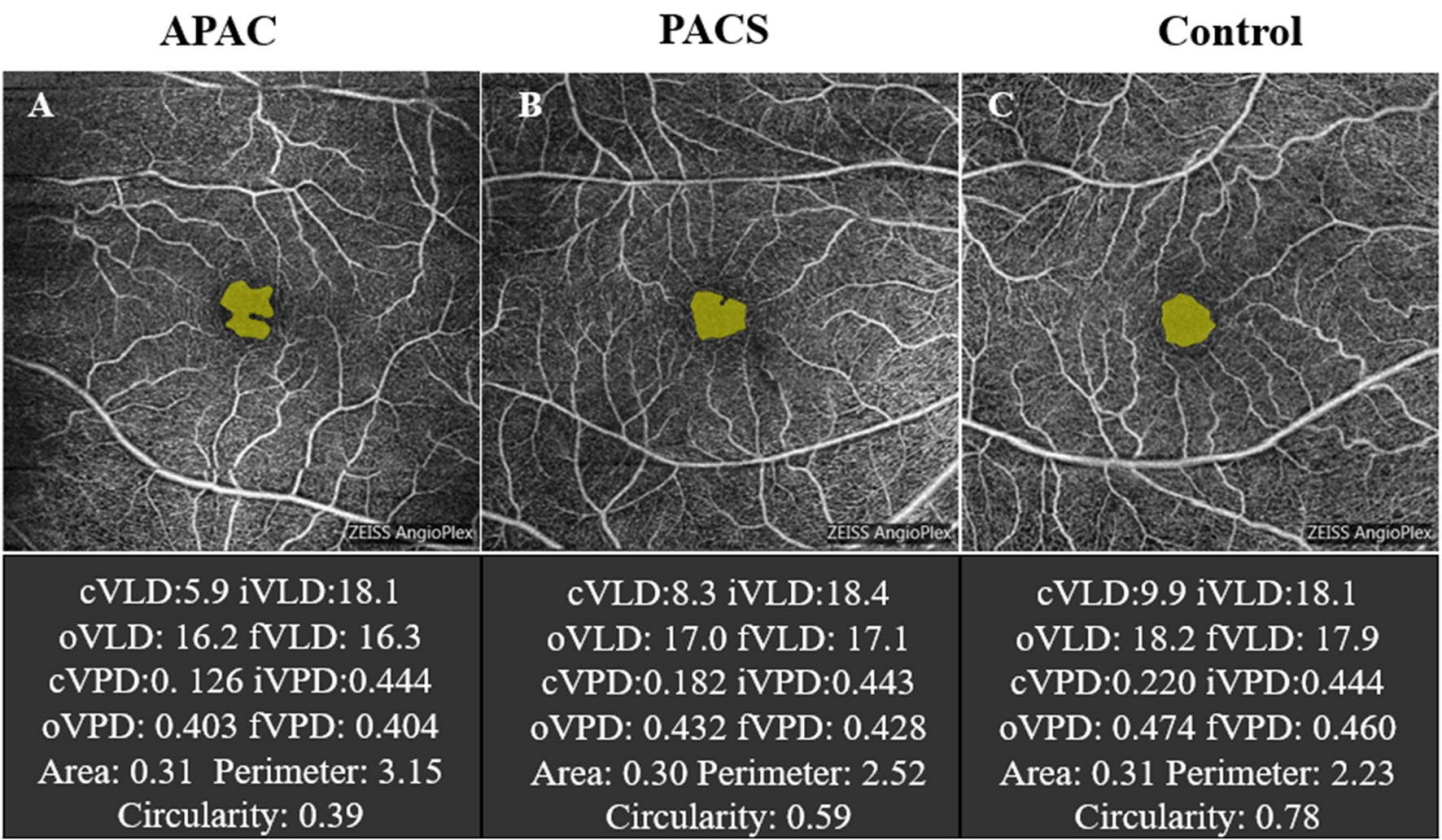
Representative cases with different macular VD and FAZ metrics. (**A**) An APAC eye has the lowest circularity index and irregular shape. (**B**) A PACS eye has the higher circularity index than APAC eyes and distorted FAZ shape. (**C**) A control eye has highest circularity index and regular shape. *APAC* acute primary angle closure, *PACS* primary angle closure suspect, *cVLD* vessel length density in central macular region, *cVPD* vessel perfusion density in central macular region, *iVL*D vessel length density in inner macular region, *iVPD* vessel perfusion density in inner macular region, *oVL*D vessel length density in outer macular region, *oVPD* vessel perfusion density in outer macular region, *fVLD* vessel length density of all regions, *fVPD* vessel length density of all regions, *FAZ* foveal avascular zone.

Figure 3 presents three representative cases showing macular VD and FAZ metrics in (A) APAC, (B) PACS, and (C) control eyes.

## Discussion

Recent OCTA studies have demonstrated VD dropout in glaucomatous eyes, such as POAG, NTG and PACG^4,10,25,26^. For PACG eyes, the pathogenesis is different from other types of glaucoma^27,28^; thus, circulation alterations may be different in the development of this disease^3,11^. But few studies have revealed vasculature loss in PACG. In this study, the characteristics of macular VD and FAZ were quantitatively analyzed and compared between APAC, PACS and normal eyes. We found that the macular VD and FAZ-related parameters in APAC eyes changed after an APAC episode compared to the parameters of PACS and normal eyes. Additionally, differences between PACS and normal eyes were detected, although there was no change in RNFL thickness or VF in PACS eyes compared to normal eyes.

Some investigations have indicated that anterior segment ischaemia caused APAC and that retinal ischaemia may be present after an APAC episode^29^. In addition, Ma et al. found that macular and papillary VD decreased significantly in eyes with narrow anterior chambers after an acute IOP elevation recently^30^. Therefore, the dropout of macular VD in APAC eyes may be due to the elevated IOP. Secondary degeneration^31^ is a mechanism to explain the reduction of RNFL thickness and VF loss after IOP rises, and Aung et al. found a significant reduction of RNFL thickness from 2 to 16 weeks after APAC^32^. However, the average duration of IOP exposure for APAC eyes was 2.35 ± 1.50 days in our study. A possible hypothesis regarding the same RNFL thickness between APAC and PACS eyes may be that retinal oedema affects the RNFL thickness after an acute attack in APAC eyes^13^. Previous reports showed that APAC eyes had a lower peripapillary VD than unaffected eyes even when IOP was normalized at 1 week after an acute primary angle closure episode, and the peripapillary VD continued to decrease for 6 weeks^12^. Next, it’s worth to study the macular VD and other structural retinal changes in PACG after treatment with anti-glaucoma surgery in future research.

The VF was decreased significantly in APAC eyes, but not in PACS eyes compared with normal eyes using the Swedish Interactive Threshold Algorithm 24-2 VF strategy. Here, we found that the central VLD was related to VF weakly in APAC eyes. Only 26.5% of the central VLD variation may have been attributed to the joint variations in VF and SBP in APAC eyes by conducting a multiple linear regression analysis. Previous studies recommended using a 10-2 strategy with glaucoma patients for detecting the central visual field, which is more closely interrelated with vision-related quality of life^33^. Besides, alteration of FAZ circularity was associated with the presence of central visual field defects in glaucoma patients^19^. Therefore, further investigations are still needed to observe the central visual field damage on the 10-2 test in APAC and PACS eyes.

From a pathophysiological point of view, PACS and normal eyes are different even though they have the same RNFL thickness and VF. Usually, PACS eyes are recognized as having shallow anterior chambers and narrow anterior chambers with or without a short axial length^34^. Some people have evidenced that early glaucomatous damage involves the macula^35^. We explored the difference between PACS and normal eyes in macular VD and demonstrated that the VLD and VPD of PACS eyes were lower in the central region. This may suggest vascular impairment before nerve injury in the parafoveal area, and parafovea VD may be an indicator for PACG diagnosis at an early stage. Retinal VD was reported to be associated with OPP, especially during the development of glaucoma in a previous research^36^. Beak et al. also described that the^23^ diurnal changes in OPP, IOP and retinal VD were greater in glaucomatous eyes than normal eyes. However, here we observed that only the inner, outer and full VPD in PACS eyes were positively correlated with OPP. We established two equations to predict macular VD in PACS eyes. These two equations showed that although RNFL thickness, DBP and OPP had combined effects on the outer and full VPD, both DBP and OPP had a smaller impact on the VPD variations than RNFL thickness. In further study, the diurnal variations in OPP, DBP, SBP and IOP should be observed in larger samples in order to gain a better understanding of the pathogenesis of PACG.

The FAZ is a central round 400 μm area devoid of retinal capillaries in the fovea, whose morphology relates to many pathologic conditions^17^. The area and regularity of the FAZ are significantly correlated with visual acuity and could be used as a diagnostic and prognostic index in diabetic retinopathy and retinal vein occlusion^19,37,38^. These researches declared that a diminished FAZ metric could be a biomarker in diseases with vascular maculopathy. To our knowledge, there is no literature about the FAZ parameter in PACG. Our investigation proven that there was no difference in FAZ area between these three groups, while APAC eyes had a smaller FAZ perimeter than control eyes, but it was not smaller than that of PACS eyes. The FAZ circularity index was highest in normal eyes and lowest in APAC eyes. In previous studies, the diagnostic ability of the FAZ area measured by conventional fluorescein angiography (FA) and OCTA was not approved for glaucoma^39^. The FAZ circularity index was applied to measure the shape relative to a circle. The index indicates an irregular shape when close to 0, and a circular shape when close to 1^19^. Geometrically, it is possible for different shapes to have the same area and perimeter according to Heron’s formula. While on the aspect of anatomy, there is only a single layer of vascular support in the FAZ, and the subtle change in the vascular arcade may render an irregular outline of the FAZ. The FAZ circularity index may be more sensitive than the FAZ area and perimeter in detecting subtle changes in the vascular arcade, and it is therefore plausible that we can detect a decreased circularity index in APAC and PACS eyes. Additionally, Choi et al.^40^ reported POAG eyes with an increased FAZ perimeter and decreased FAZ circularity index compared with control eyes, which is similar to our finding. It may suggest that there are similar pathophysiological processes in advanced POAG and PACG; accordingly we will explore the differences in FAZ parameters in different types of glaucoma in the following research. Our investigations showed that the FAZ circularity index was positively correlated with RNFL thickness in an APAC eye. Hence, we hypothesize that the reduction in RNFL thickness may be relate to microcirculatory alterations in macula. Although we can’t know whether the microcirculatory or structural alterations appear first during the development of PACG, the FAZ metric and macular VD could help us to understand the pathologic and physiological processes in PACG better.

In the current study we examined the diagnostic accuracy of all 11 parameters with AUROC. By corollary, macular VD has great diagnostic value. However, our analysis demonstrated that the FAZ circularity index was the best parameter for discriminating between PACG and control eyes (AUC 0.944), and between APAC and control eyes (AUC 0.881), with both demonstrating better AUC, sensitivity and specificity. The diagnostic efficacy of macular VD in distinguishing an APAC or PACS eye from a normal eye was significantly worse than that of the FAZ circularity index. In addition, we found that central and inner VLD and VPD had better diagnostic ability than outer and full VLD and VPD in distinguishing PACS eyes from normal eyes. All these data suggested the subtle loss of capillaries of the parafoveal vascular arcade might render an irregular outline of the FAZ prior to the decrease in macular VD outside the fovea at the early stage of PACG. Therefore, the fovea, parafoveal macular VD and FAZ circularity index may not only be an additional diagnostic approach for PACG eyes after an attack but may also help in the recognition and management of PACS patients without a history of attack.

Our study has some limitations. Firstly, this is a retrospective study with a relatively small sample of 33 subjects. Secondly, we did not examine the deep retinal and choroidal vasculature of our subjects, as those with PACG eyes had decreased VD^41^. Thirdly, all the APAC eyes were examined after fully resolving with medications, but we could not distinguish the influence of medications.

In conclusion, the changes in macular VD and FAZ parameters in APAC and PACS eyes were described compared to those of healthy control eyes using OCTA when IOP was controlled after an acute attack. APAC eyes had a progressive reduction in macular VD and FAZ circularity index, while PACS eyes presented a dropout of VD in the central macular region and FAZ circularity index. Besides, the loss of VD in the central and inner macular regions and the lower FAZ circularity index were confirmed in eyes with APAC compared with PACS eyes. Non-invasive macular assessment with OCTA could provide an accurate diagnostic tool for PACG, and the FAZ circularity index may be a potential biomarker in detecting vascular damage at an early stage in PACG eyes.

## Data Availability

Datasets from the current study are not publicly available due to compliance to privacy. Summary statistics are available from the corresponding author on reasonable request.
